# Recurrent Nausea and Vomiting in a Pregnant Woman with Chronic Marijuana Use

**DOI:** 10.1155/2018/9746062

**Published:** 2018-09-16

**Authors:** Hyunyoung G. Kim, Jeremiah Moon, Heather Dixon, Paul Tullar

**Affiliations:** Texas Tech University Health Sciences Center, School of Medicine, Department of Obstetrics and Gynecology, 1400 South Coulter Street, Amarillo, TX 79106, USA

## Abstract

**Background:**

Cannabinoid hyperemesis syndrome is a condition characterized by chronic cannabis use and cyclic episodes of nausea, vomiting, and abdominal pain, relieved by compulsive bathing. The syndrome is likely to be underdiagnosed in pregnant women due to its similarity with hyperemesis gravidarum in the presentation.

**Case:**

We report a 20-year-old pregnant woman with multiple admissions for recurrent nausea and vomiting who was observed to be taking frequent hot showers. Without other identifiable causes, she was diagnosed with cannabinoid hyperemesis syndrome and managed with antiemetics and abstinence.

**Conclusion:**

Abstinence from cannabis use is highly recommended in pregnant women due to its potential harm in fetal development and stimulation of intractable nausea and vomiting. Recognizing the symptoms and proper history taking prompt early diagnosis, allowing timely and adequate treatment.

## 1. Introduction

Marijuana is the most commonly reported illicit drug used during pregnancy, with estimated prevalence of 2-5% in some studies and as high as 15-28% in young, urban low socioeconomic women [1]. With recent legalization of marijuana in several states in the United States, the prevalence of marijuana use during pregnancy is expected to rise.

Chronic and heavy marijuana use can lead to a condition called cannabinoid hyperemesis syndrome (CHS). It presents with symptoms of recurrent nausea, vomiting, and abdominal pain that are temporarily relieved with hot bathing [2–5]. While the syndrome can also arise during pregnancy, diagnosis of CHS is often delayed due to the vague symptoms of nausea and vomiting, which also occurs in early pregnancy and hyperemesis gravidarum [2].

The combination of severe vomiting, nausea, and frequent hot showers can lead to serious complications such as volume depletion, weight loss, and esophageal rupture [3]. Furthermore, frequent hot showers over long periods of time may increase risk of fetal neural tube defects, gastroschisis, and omphalocele [6]. Increased body temperature from these hot showers may also be associated with preterm labor and epilepsy as well [7–9].

## 2. Case

A 20-year-old woman, gravida 7 para 0060, at 14+3/7 weeks gestation by 8-week ultrasonography with history of depression and bipolar disorder presented to the emergency department (ED) complaining of unremitting nausea and vomiting, exacerbated by oral intake, for the past several weeks. She also reported intermittent hematemesis, epigastric abdominal pain, and mild diarrhea. However, she denied headache, fever, chills, chest pain, shortness of breath, palpitations, or dizziness. She also denied vaginal bleeding and discharge, vaginal bleeding, leakage of fluid, contractions, suprapubic or pelvic pain. She was given IV hydration, ondansetron, famotidine, and metoclopramide but continued to vomit without visible blood. She had mild hypokalemia, which was replaced with intravenous (IV) and oral potassium. She slowly improved throughout her hospital course and was able to tolerate oral intake by hospital day 6. The patient had a two-year history of multiple ED visits for the same issue, for which she was diagnosed with hyperemesis gravidarum and managed with hydration and antiemetics.

During the following month, the patient returned several times with the same complaints. She was treated again with IV hydration and antiemetics (oral ondansetron and promethazine suppositories), which resolved her symptoms. She was instructed to continue her home doxylamine succinate-pyridoxine hydrochloride and prescribed ondansetron and promethazine prophylactically.

At 22+4/7 weeks gestation, the patient presented to labor and delivery again with complaint of nausea and vomiting. She reported that she remained free of symptoms for approximately a month with home medications but began vomiting again with inability to keep fluids or solids down for the past 12 hours following consumption of contaminated food. She denied any fever, chills, diarrhea, headache, or blurred vision. She reported good fetal movement and no vaginal bleeding or discharge or contractions. While on the labor and delivery, she continued to vomit with antiemetics (IV ondansetron and promethazine). On the hospital day 2, patient was found in the shower and reported that warm showers are the only relieving factor for nausea and vomiting—concerning for CHS. Urine drug screening (UDS) was performed and was positive for cannabinoids. The patient was informed that this was possibly related to her cannabis exposure. She remained abstinent throughout the hospital stay and was continued on IV fluids and antiemetics. On the hospital day 3, she noted vast improvement and was able to tolerate regular diet. Patient was discharged home with promethazine, ondansetron, and doxylamine succinate-pyridoxine hydrochloride. Patient was counseled on completely discontinuing all exposure to cannabis and voiced understanding. UDS remained negative at subsequent prenatal visits.

At 40+1/7 weeks gestation, the patient delivered vaginally a live female infant weighing 3.19 kg with APGAR score of 8/9 without any complications. The mother and baby were discharged home on the second postpartum day.

## 3. Discussion

While marijuana use in pregnancy is expected to rise due to increase in its liberalization and popularity, there has been lack of studies on its pharmacokinetic effect on pregnant women and their fetuses. The current preliminary data suggest that delta-9-tetrahydrocannabinol (THC), a main active ingredient in marijuana, crosses the placenta, and the prenatal exposure to THC may negatively impact the child's future higher cognitive functions as well as psychological development [10–14]. Therefore, it is recommended that pregnant women refrain from marijuana use until further information is available and proven otherwise.

Although cannabis is usually known for reducing nausea and vomiting, various theories have been proposed to explain the pathophysiology of CHS. While THC produces an antiemetic effect by activating the G-protein coupled cannabinoid 1 (CB1) receptors in the dorsal vagal complex, some suggest its greater effect on the CB1 receptors in the enteric nervous system, which decreases peristalsis and gastric emptying, inducing emesis [15]. There is also an evidence that synthetic marijuana may cause CHS by overactivating the CB1 receptors [5]. In addition, THC's tendency to sequester in fat tissue may explain why chronic, heavy users of marijuana are more prone to CHS [16]. On the other hand, other nonpsychoactive components of marijuana, such as cannabidiol and cannabigerol, may also have a similar correlation with vomiting. In their study, Parker et al. showed that cannabidiol acts as an antiemetic in low levels and proemetic in higher levels in shrews [17].

As seen with our patient, frequent hot bathing has been reported to relieve symptoms of nausea and vomiting in CHS. Among several theories to explain this interesting response, one proposes that increased body temperature may turn off chronic hypothalamic activation by cannabinoids. Another theory posits that dilation of cutaneous vessels may decrease blood flow to the cannabinoid-vasodilated splanchnic vessels, which may reduce nausea and vomiting [4]. Regardless of the mechanism, frequent hot bathing may exacerbate dehydration, leading to hypotension and increased risk of falls. Increased dehydration with heat exposure diverts blood flow to the skin, away from the maternal organs and fetus, and increases antidiuretic hormone and oxytocin release, inducing preterm labor [8]. Moreover, consequent maternal weight loss further increases the risk of preterm labor and small gestation for age [18]. Maternal use of hot tubs for over 30 minutes during the first trimester—which many patients with CHS greatly exceed—has also been associated with neural tube defects, esophageal atresia, omphalocele, and gastroschisis [6].

Dehydration is a common consequence of repetitive nausea and vomiting, and therefore, fluid resuscitation is usually required in the CHS patients [3, 4]. In alleviating symptoms of CHS, traditional antiemetic medications such as ondansetron, promethazine, and metoclopramide have been reported to be fairly ineffective. Alternatively, benzodiazepines, haloperidol, and capsaicin have been shown to be effective in the management of acute and tricyclic antidepressants in that of chronic CHS [19]. Although traditionally used in treatment of psychosis, haloperidol has an effect on dopamine D2 receptors in the chemoreceptor zone and potentially on cannabinoid 1 (CB1) receptors in the central nervous system. In a report by Hickey et al., IV haloperidol (5 mg) was administered in a patient with CHS, and he reported relief of symptoms within one hour with no vomiting for the subsequent eight hours [20]. Topical capsaicin had been explained to produce an antiemetic effect by binding to the transient receptor potential vanilloid 1 receptor, located near CB1 receptors throughout the body, and inhibiting the release of substance P, involved in nausea and vomiting [21, 22]. While our patient was managed with traditional antiemetics, earlier diagnosis of CHS and treatment with benzodiazepines or haloperidol could have led to faster resolution of symptoms. Future prospective trials are needed to evaluate and define optimal pharmacologic treatment of CHS.

The best treatment is, nevertheless, cessation of marijuana use, which could take up to a week for noticeable improvement [2]. Patients often attempt to treat their symptoms with more marijuana consumption, which can worsen or prolong their episodes of emesis [4]. Therefore, education, counseling, and cognitive behavioral therapy can be crucial components in successful treatment and prevention of these episodes.

## 4. Conclusion

Chronic or excessive marijuana use during pregnancy can induce CHS and poses potential health risks on both the mother and fetus. Our case of CHS was primarily managed with antiemetics and cessation of cannabinoid use despite the well-documented resistance to traditional antiemetic treatment. While benzodiazepines, haloperidol, and capsaicin have been reported to be effective, further studies are necessary to establish the optimal management of CHS. The diagnosis of CHS in pregnancy can be challenging as it presents with repetitive nausea and vomiting, which overlap with symptoms of hyperemesis gravidarum in early pregnancy. Awareness and recognition of this syndrome will prompt early and appropriate management, avoidance of unnecessary workup and cost reduction, and lessening of provider frustration with inefficacious treatment.

## Data Availability

No data were used to support this study.
